# Improving remote material classification ability with thermal imagery

**DOI:** 10.1038/s41598-022-21588-4

**Published:** 2022-10-14

**Authors:** Willi Großmann, Helena Horn, Oliver Niggemann

**Affiliations:** Helmut-Schmidt-University, University of the Bundeswehr, 22043 Hamburg, Germany

**Keywords:** Mechanical engineering, Chemical engineering, Computer science

## Abstract

Material recognition using optical sensors is a key enabler technology in the field of automation. Nowadays, in the age of deep learning, the challenge shifted from (manual) feature engineering to collecting big data. State of the art recognition approaches are based on deep neural networks employing huge databases. But still, it is difficult to transfer these latest recognition results into the wild—various lighting conditions, a changing image quality, or different and new material classes are challenging complications. Evaluating a larger electromagnetic spectrum is one way to master these challenges. In this study, the infrared (IR) emissivity as a material specific property is investigated regarding its suitability for increasing the material classification reliability. Predictions of a deep learning model are combined with engineered features from IR data. This approach increases the overall accuracy and helps to differentiate between materials that visually appear similar. The solution is verified using real data from the field of automatized disinfection processes.

## Introduction

Humans recognize materials based on spectral, texture, and context data^1^. Machine vision simulates this cognitive process in many industrial applications. Application areas for material recognition are, for example, sorting processes such as waste separation^2^, the monitoring of construction progress^3^, and urban or botanical investigations with remote sensing^4^. The knowledge about material properties is of great importance for the interaction of robots with everyday objects^5^ or for the ongoing automation of manufacturing and other industrial processes using modern smart technology, also known as Industry 4.0^6^.

Due to the coronavirus pandemic, the development of automatized disinfection processes has recently become a field of special interest. The recognition of materials is highly important in these applications, because material properties influence the persistence of pathogens^7^ as well as the effectiveness of the disinfectant^8^. An incorrect material classification could lead to an incorrect application of disinfectant and thus to an insufficient disinfection process.

One contemporary approach of material recognition uses Convolutional Neural Networks (CNN) to base identification not only on the consideration of different kind of visual data but also on created and learned context between given information. Huge existing material databases^9–11^ enable the training of deep CNNs and allow investigations of material recognition possibilities on the basis of images. Additionally, a broad range of pretrained CNNs is available for transfer learning, giving the advantage of less effort to build databases and to train CNNs for new applications. By using such pretrained CNNs, less data is needed to train a solution for specific application scenarios^12^.

However, the material classes of existing databases are defined too general for many technical applications. In disinfection or sorting processes, for example, the detection of various metallic materials or the distinction between wood and wood imitation, is a basic requirement. This distinction is much more difficult to determine because visually similar materials such as aluminum and stainless steel need to be considered.

Therefore, using only CNNs to evaluate visual appearance of materials no longer seems to be the solution. Even CNNs cannot distinguish between similar visual data and thus between materials with the same colors and textures.

A possible solution to this problem could be the evaluation of a larger electromagnetic spectrum, because the material specific information in the data increases. In this case, the identification could be almost 100 % precise, but this would require relatively expensive measuring equipment.

As an alternative solution, the use of the IR range seems to be a good compromise because it requires rather inexpensive cameras for the detection of thermal radiation^13^. Based on these circumstances, this study follows three research hypotheses (RH):

RH 1: Evaluating the IR range additionally to the VIS range is a cost-effective option to significantly improve the reliability of remote material recognition for industrial processes.
RH 2: In a controlled environment, thermal imaging helps to differentiate between materials that visually appear to be similar. According to the Stefan-Boltzmann law, the emissivity of technical bodies influences their thermal radiation performance. Therefore, the apparent temperature in relation to the true body temperature leads to the infrared emissivity as a material specific property.
RH 3: The median and the variance of the relativized material temperatures are characteristic material features. Recognition accuracy can be increased when these engineered features are combined with learned VIS features from a CNN. A support vector machine (SVM) is suitable for this feature fusion. This approach outperforms other methods from the field of remote sensing such as data level fusion or image fusion.

The contributions of this paper are briefly described as follows.

(i) Material classes with a high technical range of application are differentiated. A distinction is made between different metals and visually similar materials.
(ii) For this purpose, the material specific apparent temperature is fused with learned VIS features to increase the recognition accuracy for industrial remote sensing applications.
(iii) The proposed fusion algorithm outperforms visual material recognition approaches and comparable sensor fusion algorithms from the field of remote sensing.

The rest of this paper is organized as follows: At first, related literature regarding material recognition approaches, thermal imaging, and electro optical sensor fusion is presented (In "Related work" section ). Next, details for the proposed architecture (In "Solution" section) and for the experimental design of the new material recognition approach (In "Experiments and results" section ) are provided. Finally, concluding remarks and further investigations (In "Conclusion and outlook" section) are proposed.

## Related work

Early material recognition approaches evaluated color distributions and patterns. Adelson^14^ started to study the general perception of materials and suggested differentiating materials based on their reflectivity and shape. Liu et al.^15^ suggested an extended rating to cover more aspects of appearance. They found that reflectance, texture, color, shape, and environment illumination are suitable parameters for material classification.

Experimenting on the Flickr Material Database (FMD)^9^, Badami^16^ found a SVM is better suited to classify these features than previous methods. FMD therefore covers many aspects of the appearance of different materials.

Sharan et al.^17^ confirmed these results with their investigations and showed that local image information such as color, texture, or shape are not sufficient for material recognition. For better classification results they suggested taking context information (like the object category) into account.

Convolutional neural networks do not require any local image information to be given for material classification. Instead, the algorithm learns the features required for recognition. Indeed, modern methods using CNNs show significantly better results^10,18^.

The disadvantage of learning these networks from scratch is the high demand for labeled data. Possibilities to reduce this high demand for data are the employment of pretrained models^19,20^.

Recent studies apply ensemble learning approaches to combine multiple pretrained classifiers^11,21^ and achieve better classification results. However, higher accuracies can also be achieved by evaluating more specific material data with less computationally intensive algorithms. The latter could be addressed by the evaluation of IR data in addition to the visual information of the materials.

So far, only a few studies take the IR range into account. Its suitability for material classification has been shown in laboratory environment^22^. Based on a thermodynamic model, they examined the material specific heat conduction for classification using a Nearest Neighbor algorithm. Additional parameters are based on a water permeation experiment. However, the experimental setup is not designed for industrial use. The material samples are heated, moistened, and recorded from a short distance for several minutes.

Another approach also considers changing environmental conditions^13^. Thermal images are taken into the wild and classified using a CNN. However, the recording distance stays constant at approximately 0.5 m and the materials are chosen with quite characteristic patterns. Erickson et al.^23^ evaluate eight material classes of everyday objects. Here, the material recognition is improved by additional IR images in order to optimize the gripping process of a robot.

A more recent approach exploited thermal conductivities by making physical contact and observing temperatures during heat transfer^24^. This research confirmed that evaluating thermal data improves the accuracy of material identification.

**Figure 1:**
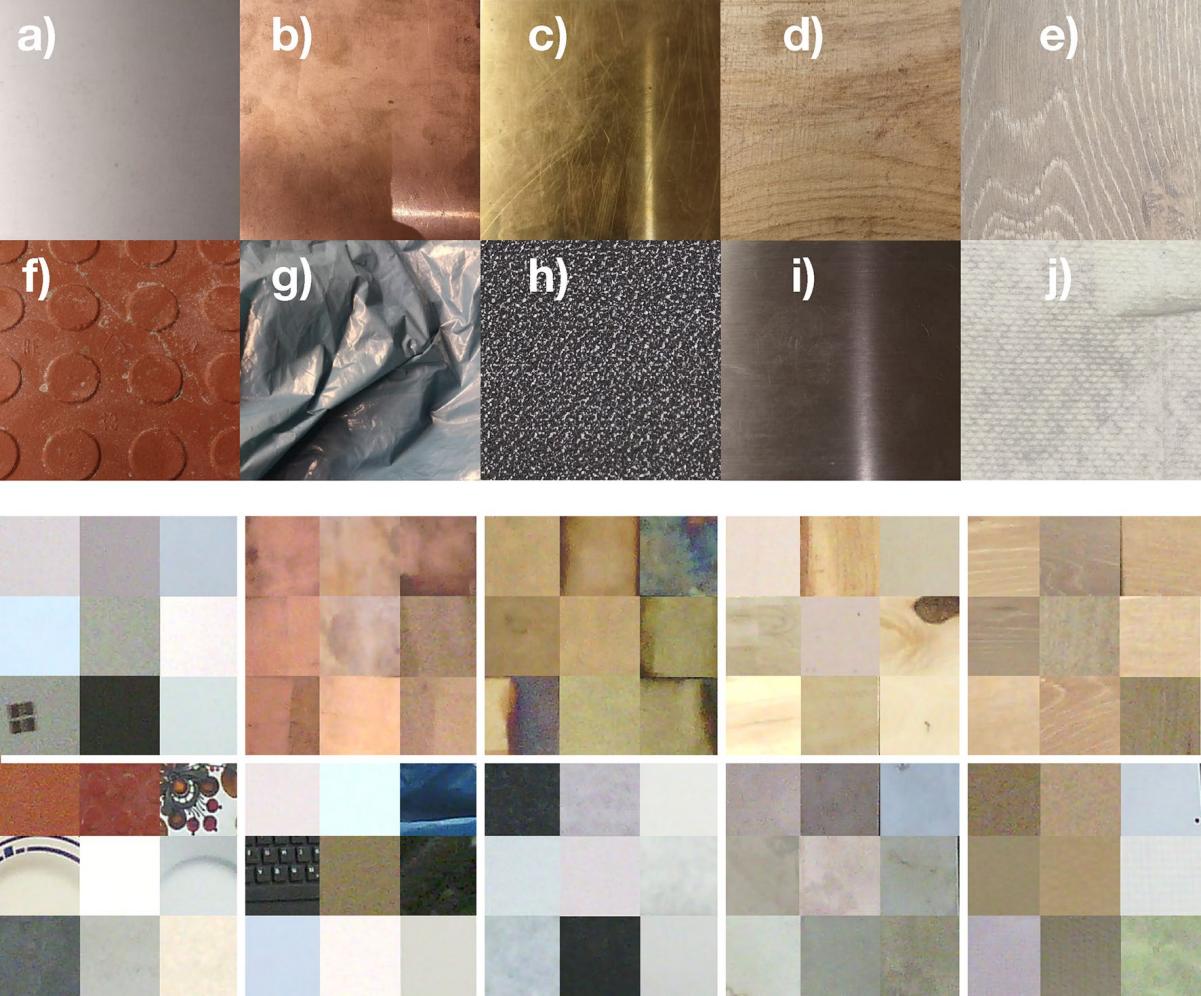
Material classes and samples of the database used. The first two rows show some material samples: (**a**) Aluminum, (**b**) Copper, (**c**) Brass, (**d**) Wood, (**e**) Wood Imitations, (**f**) Ceramics, (**g**) Plastics, (**h**) Textiles, (**i**) Stainless Steel, (**j**) Cellulose. Some cropped images are shown in the same order in the lower two rows. These images were used for training and evaluation of the CNN.

While an IR- and VIS data fusion is not applied in most approaches for material recognition, it is quite commonly needed for electro-optical systems in different areas of remote sensing. State of the art approaches apply image fusions within a fusion network^25^. For example, Li et al.^26^ propose a VGG 19 framework for an image fusion of RGB and IR images on feature level. Xu et al.^27^ introduce an unified unsupervised network for multi-sensor, multi-exposure, and multi-focus image fusion tasks. Xiao et al.^28^ are focusing on infrared - visible image fusing by introducing a Teacher-Student Network. This enables the fusion of multi resolution images.

However, these fusion approaches do not meet the requirements of this work. The aim of image fusions from the field of remote sensing is the combination of complementary pattern information from several source images. The material data available here show few distinctive temperature and visual patterns. In addition, since images are fused, these approaches do not consider combining temperature and visual data.

Remote sensing applications required for autonomous driving successfully apply SVMs for multimodal sensor fusions^29^. These applications need to fuse visual images with multiple disparate data such as radar or vibration information^30^.

Therefore, in order to use material recognition algorithms for industrial applications, several additional investigations are required. Based on the research hypotheses, this study examines the following circumstances that have not been taken into account yet:

- (RH 1) The previous material recognition approaches focused on the investigation of general visual phenomena. The material classes were selected based on specific patterns or general appearance. The material data of this study focuses on high technical benefit and a wide range of applications. In addition, different lighting conditions and shooting distances are considered to take realistic environmental conditions into account for industrial applications.
- (RH 2) The basic feasibility of using thermal data for material recognition has already been proven. However, the recognition of visually similar materials or imitations has not been investigated yet. Everyday materials often appear similar. For example, plastics can be used as an imitation for metals or wood. In order to evaluate this, a distinction is made in this study between visually similar material classes such as aluminum and stainless steel or wood and wood imitation.
- (RH 3) SVMs are a proven algorithm for classifying engineered and learned features. They are also used successfully to evaluate various sensor data from electro-optical systems. However, it must first be evaluated whether the measured temperature distributions have material specific properties. From this distributions, features can be engineered that help with material identification.

## Solution

Since there is no known database that combines IR and VIS for the required material classes, a new database is specifically created for this study. The VIS feature extraction and evaluation is done with a CNN, while the final sensor fusion is realized using a SVM. The proposed algorithm fuses learned VIS features with engineered IR features. This enables the identification of visually similar materials.

This study focuses on raw temperature data instead of false color images. According to the Stefan-Boltzmann law, the temperature data appear comparatively characteristic. This enables a description with statistical parameters. The material specific expected temperature value is estimated by calculating the median of a temperature field. The median is chosen instead of the average because of its greater robustness to outliers. In addition, the variance around mean of the temperature is taken as a scatter parameter.

The visual data appear significantly less characteristic. This data is evaluated and classified here using a CNN. For the proposed solution, the results from the CNN are combined with the engineered statistical parameters.

### Database

Aluminum, copper, brass, wood, wood imitations, ceramics, plastics, textiles, stainless steel, and paper are chosen for evaluation. These materials are broadly applied in industry and have certain similarities in texture and color.

The images of the materials are presented in Fig. 1, showing the resemblance between brass (c), wood (d), wood-imitations (e) and cellulose (j) as well as between aluminum (a), stainless steel (i), and some of the textiles (h).

**Figure 2:**
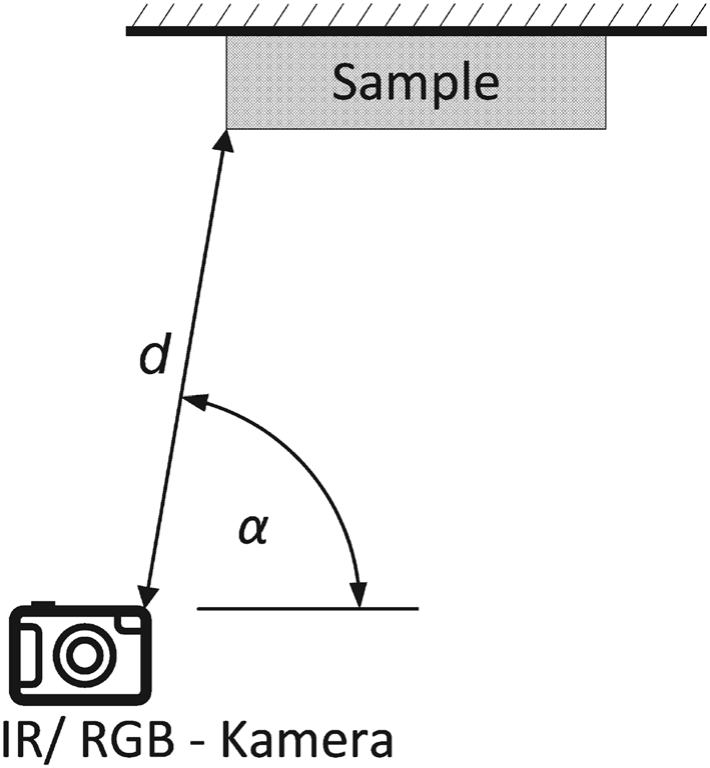
Experimental design of collecting images from material samples: The IR and RGB images were taken with an angle $\alpha < 90 \ ^\circ$ and a distance *d* between 1 and 2.5 m.

**Figure 3:**
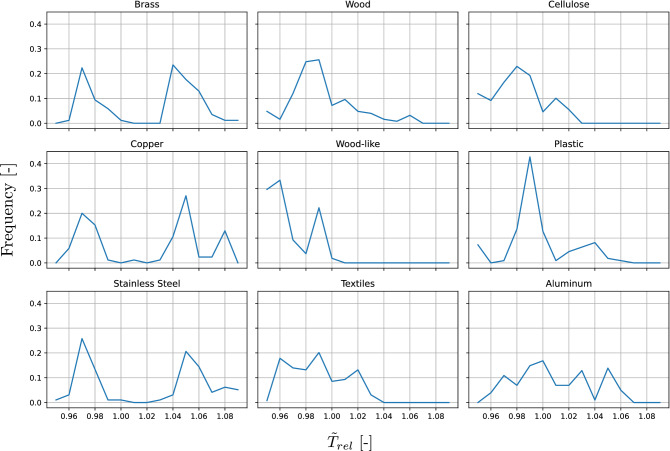
Histograms of the material specific median relative temperature $\tilde{T}_{rel}$. As described in Algorithm 1, the temperature fields of each material sample were put into perspective with the respective ambient temperature. The median was then calculated for each temperature field. The distribution across the database is presented in the histograms above.

The material samples are photographed from distances $d \in \{1, 1.5, 2, 2.5\}$ m with an angle $\alpha < 90 \ ^\circ$, as presented in Fig. 2.

The samples are placed on cardboard to facilitate segmentation. The pictures are taken with the FLIR T540 (Flir Systems Inc., Wilsonville, OR, US). This uncooled thermal camera has an integrated digital camera which enables taking IR and RGB images simultaneously.

The database includes 1112 cropped RGB images with corresponding temperature distributions. The images are taken indoor in closed rooms avoiding thermal radiation reflections and ensuring the sample temperature corresponded with the ambient temperature. On this account, the material specific IR emissivity leads to material specific temperature distributions, when measured with the thermal camera.

### Feature extraction with relearned CNN

Cropped images from the database are used to relearn a VGG 16^31^. This CNN is pretrained on the Imagenet^32^ database and loaded from the PyTorch Model Zoo. The VGG 16 architecture itself has proven suitable as a classification model for material recognition applications^3,10,18^.

**Table 1:** Applied VGG 16 configuration.

VGG 16	
Features	Classifier
2 x Conv2d - 64	FC - 4096
Maxpool	Dropout - 0.5
2 x Conv2d - 128	FC - 4096
Maxpool	Dropout - 0.5
3 x Conv2d - 256	FC - 10
Maxpool	Softmax
3 x Conv2d - 512	
Maxpool	
3 x Conv2d - 512	
Maxpool	

All 2d convolution kernels have a receptive field size of 3*x*3 pixels. A ReLU activation function is applied after the Convolution- (Conv2d) and Fully Connected layers (FC). During training two Dropout layers randomly zeroes some FC elements with a probability of 0.5. Finally, the output $\pmb {p}$ is calculated by applying a Softmax activation function.

The cropped material images as shown in Fig. 1 show very few characteristic patterns and many color similarities. Compared to the Imagenet database, better classification results cannot be achieved by learning more complex features such as object shape. The recognition ability of visually similar materials is instead improved by using IR data. That is why the proven feed-forward VGG 16 is suitable as the baseline model.

For transfer learning, the prediction layer is replaced with an adapted fully connected layer. While the original VGG 16 architecture can classify 1,000 different labels, only 10 are required here. The relearned CNN, as shown in Table 1, is then used to classify the RGB images from the database. The applied hyper parameters are presented in Table 2. Applied data pre-processing includes pixel standardization and image resizing to $224 \times 224$ pixels.

Finally, if the cropped image $I_\text {RGB} \in \mathbb {R}^{83 \times 83 \times 3}$ is the input and $\pmb {p} \in \mathbb {R}^{10}$ are the outputs of the classification model $CNN(\cdot )$, the above relation can be formally written as $\pmb {p} = CNN(I_\text {RGB})$. The Softmax outputs are used here instead of the raw CNN features due to the much higher information density.

**Figure 4:**
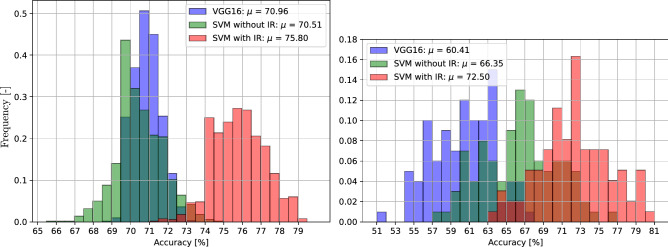
Histograms of the convergence analysis: To evaluate whether the algorithm converges, one experiment was done 1,000 times with a constant train test split (left) and another with 10 times in a stratified tenfold split (right).

### Sensor fusion with SVM

**Figure 5:**
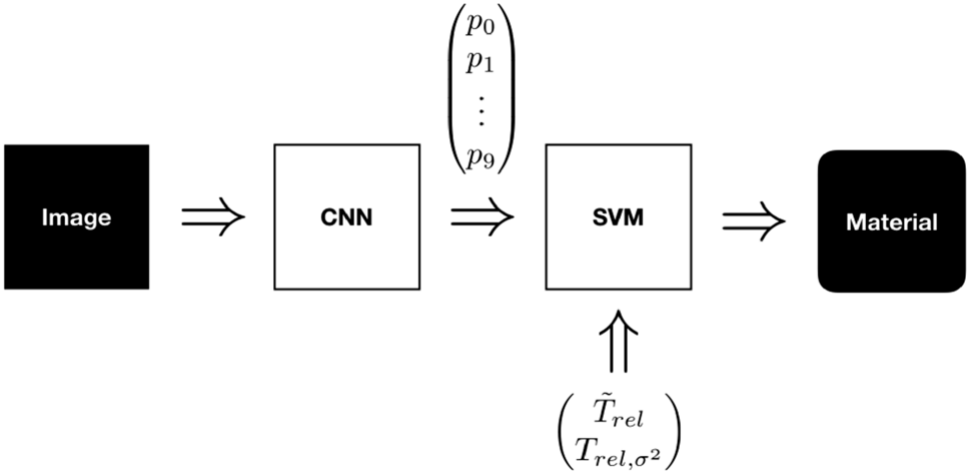
Algorithm pipeline: The CNN extracts and classifies VIS features from a RGB Image. The Softmax outputs $\pmb {p}$, the median relative temperature $\tilde{T}_\text {rel}$ and the variance $T_{\text {rel}, \sigma ^2}$ are part of the feature vector. A SVM merges these features for classification and predicts the resulting material.

The temperatures of each temperature field *T* are relativized by dividing them with the ambient temperature $\vartheta _\text {a}$. In order to consider IR data for material recognition, the median $Med(\cdot )$ and variance $Var(\cdot )$ of the relative sample temperatures are used as features and linked with the Softmax outputs $\pmb {p}$ of the VGG 16 as shown in Fig. 5. The median is preferred to the mean as the expected value, as this is more robust to outliers.

Thus, the feature vector $\pmb {x}$ consists of predictions per material class (VIS features) and median as well as variance around mean of the relative sample temperatures (IR features), what is formally presented in Algorithm 1. 
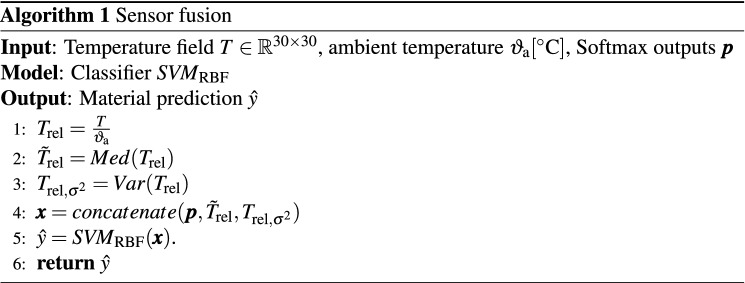


A SVM with Radial Basis Function (RBF) kernel and the one–versus-one approach for the multi-class classification $SVM _\text {RBF}(\cdot )$ is chosen to classify the materials based on the combined feature vector. Compared to other knowledge-based classifiers such as Nearest Neighbor or Random Forests, SVMs find the most robust separation of given features due to their optimization algorithm.

The proposed 2-stage design is preferred to an end-to-end solution for this study because IR features can be examined objectively. More precisely, repeated learning of neural networks leads to statistical errors. This influence is eliminated in the experiments by introducing the second stage.

## Experiments and results

Subsequently, the median relative temperature $\tilde{T}_\text {rel}$ is calculated and histograms are created, describing the frequency of the median values per class(see Fig. 3). These histograms show characteristic distributions for each material. So, in principle, the classification of materials must benefit from this additional IR data. Especially the metals shown in the first column have a very specific frequency distribution, which clearly differentiates them from non-metals and aluminum. Aluminum is covered by a characteristic oxide layer, which influences the emissivity.

### Empirical tests

For a convergence analysis, 80 % of the samples are randomly chosen for the training and 20 % for the validation. The training set is oversampled to balance the numbers of samples per class. The balanced dataset is used to relearn the pretrained VGG 16 1,000 times applying the hyperparameters shown in Table 2.

**Table 2:** Table of hyperparameters used to implement the transfer learning.

Hyperparams	
Epochs	20
Batch size	10
Optimizer	Adam algorithm
Learning rate	$1*10^{-4}$
Criterion	Cross entropy loss
Split	Stratified tenfold
Random state	42

Finally, the SVM is fitted with the Softmax outputs from the CNN and the IR features. For image processing and the subsequent evaluation, Python 3.7 specifically the packages Scikit-Image^33^, PyTorch^34^ and Scikit-Learn^35^ are used.

To investigate the influence of the IR features, an additional classification is done with dropped IR features as baseline. The resulting probability distribution of the accuracies is presented in Fig. 4, on the left side.

The VGG 16 (blue) clearly converges to the arithmetic mean $\mu = 70.96$. The mean of the SVM without IR features (green) is nearly the same as the VGG 16 while the SVM with IR features (red) is showing significant better results as the SVM with dropped IR features. A one-sided paired t-test confirms this with a p-value of one.

To further examine the algorithm on the database, a cross validation is done ten times in a second test with a stratified tenfold split of the database. The probabilities of the accuracies are shown in Fig. 4, on the right side. Except of the split ratio, here nine to one, all other model settings are the same. Even when the distributions are not as separated as in the previous test, the improvement in accuracy of material recognition when using IR features is still significant, as another one-sided paired t-test confirms with a p-value of one.

**Figure 6:**
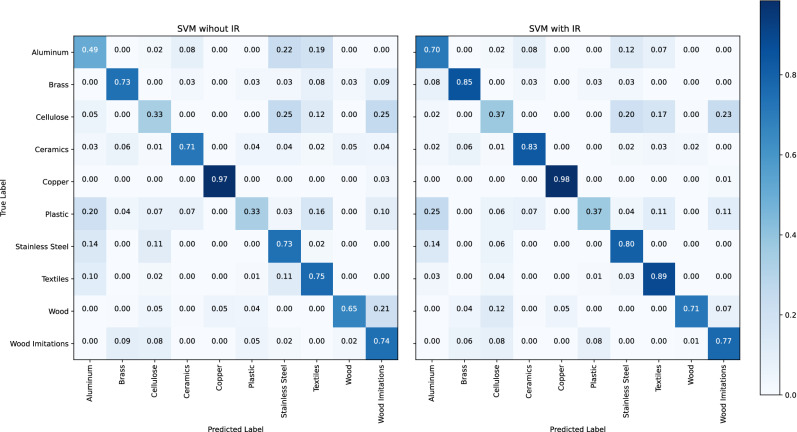
Confusion matrices from the cross validation test. The mean accuracies are shown when the feature vector is classified with dropped IR data (left) and with included IR data (right).

### Comparison to previous work

State of the art material recognition approaches use CNNs to classify visual images. The VGG 16 has proven itself and is taken here to extract the visual features. A comparison to recent classifiers is presented in Table 3.

To investigate each network capability for feature extraction, similar classifier parts (see Table 1) are chosen. In addition the hyperparameters are the same as presented in Table 2. All models are loaded pretrained on the ImageNet dataset. The classifiers of all three models are relearned as presented in "Feature extraction with relearned CNN" section.

**Table 3:** Baseline study comparing different CNNs for visual feature extraction.

Model	Precision	Accuracy	F1-Score	Rel. Time
VGG 16^31^	0.6757	**0.6321**	0.6216	1
MobileNetV3Large^36^	0.1443	0.1798	0.1072	0.77
InceptionResNetV2^37^	0.5532	0.4793	0.4772	2.95

Scores are averaged over a tenfold split and sample wise weighted to account for imbalanced data. The computing time required to evaluate a ten-fold split is set in relation to the time required for the VGG 16.

The highest accuracy value is in [bold].

MobileNets are compact CNNs designed for lightweight applications. On the other hand, ResNet models are able to learn very complex features due to their very deep depth. However, the VGG16 clearly outperforms the other approaches.

To enable the most meaningful comparison possible, all experiments apply the same classification algorithm (see "Feature extraction with relearned CNN" section) with the same hyperparameters (see Table 2). The only difference is the proposed sensor fusion.

While there is no comparable approach in the field of material detection, optical sensor fusions are often performed in similar remote sensing applications. Two approaches are presented here for comparison. The first state of the art image fusion approach proposes a fusion of super-resolution of infrared and visible images^28^. The merged image contains key features of both input images.

Since this study uses temperature arrays, a transformation is realized by normalizing and multiplying by 255. The result therefore corresponds to an 8-bit grayscale image. Infrared and visible images are aligned and rescaled.

The second approach applies an information fusion^38^. They suggest to set the grayscale IR image as an additional color channel. The fused image is classified using a relearned VGG 16 with extended first layer kernels. The new initial weights are set as the mean RGB weights. Here, the temperature array is rescaled, aligned, and standardized to implement this data fusion approach. The mean results of a tenfold split are presented in Table 4. The same setup is used for classification in all experiments.

**Table 4:** Comparison of different sensor fusion approaches.

Method	Precision	Accuracy	F1-Score
VIS	0.6757	0.6321	0.6216
IR	0.1989	0.2023	0.1637
Feature fusion^28^	0.4156	0.3588	0.3296
Information fusion^38^	0.5258	0.5323	0.4979
Proposed fusion	0.7535	**0.7347**	0.7325

The VGG 16 model is applied in all experiments to ensure comparability.

The highest accuracy value is in [bold].

The proposed fusion and the feature fusion method are both two stage approaches. But the proposed fusion clearly outperforms the other feature fusion approach. The aim of image fusions from the field of remote sensing is the combination of complementary pattern information from different sensors. However, the material data available here show few distinctive IR patterns and many visual similarities. The characteristic of the IR data is more like a material-specific bias, as shown in Fig. 3.

The other comparison relates to an early sensor fusion approach. The IR and VIS images are convolved within the first CNN layer. Although theoretically there is less information loss with this approach, actual results lag behind the proposed approach.

However, early information fusion is more accurate than the later feature fusion. This might be because color and texture from the visual image can be successfully combined with the different gray levels of the infrared image. The classification results of the visual and infrared data are given for completeness.

### Evaluation of experiments

**Figure 7:**
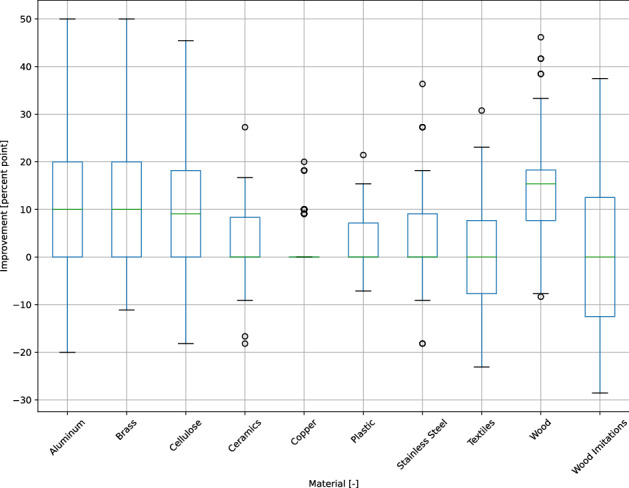
Boxplots of the material specific improvement when using IR features for ten times in a tenfold cross validation.

For cross validation, the material specific benefit of IR data as an additional feature is demonstrated in Fig. 7. The median improvement of the accuracy from aluminum, brass, and cellulose is about ten percent points (pp). Wood shows the best improvement of more than 15 pp. The median of the other materials is about zero, to be discussed next in Fig. 6.

It shows the arithmetic mean accuracy of each predicted vs. true label, comparing the classification without (left) and with (right) IR data. The SVM, using a feature vector with dropped thermal features, reaches an overall mean accuracy of 66.4 % in the sample recognition. The overall mean accuracy increases by 6.1 pp when the IR features are added.

In principle, regarding the mean accuracies per material, the combined model benefits from IR features. This is when VIS features are similar and IR features are different. The combined model can distinguish, for example, much better between wood and wood imitations by using IR data. The mean accuracy of the class wood imitations increases just by 3 pp, because more samples are classified as plastic, being the actual material.

The accuracy of aluminum recognition increases by 21 pp because the miss classification as stainless steel or as textiles decreases. The IR data helps to distinguish between these two metals, which visually appear to be similar. As shown in Fig. 3, aluminums emissivity significantly differs from the emissivity of other metals because of its characteristic oxide layer. Many of the cropped textile images visually appear similar like stainless steel or aluminum, as Fig. 1 shows. About 20 % of the textile samples are false classified as these materials. By using IR data, the mean accuracy of textiles increases by 14 pp.

The recognition accuracy of brass increases by 12 pp, because the differentiation from wood-imitations, wood, and textiles is enhanced by IR data. On the other hand, 8 pp more brass samples were classified as aluminum although VIS- and IR features should be different. This could be an indication to train the CNN within further epochs.

Plastics and cellulose are the least recognized material classes with an accuracy of 33 % without IR data. Their recognition increases by 4 pp when IR data is considered. It seems like the CNN was not able to learn the characteristic visual properties. Additionally, 10 % of the plastics were classified as wood imitate, which actually is plastic.

Copper shows no improvement, see Fig. 7. That is because the accuracy with dropped IR features is already at 97 %. However, the mean accuracy can still be improved when IR features are added, as Fig. 6 shows.

IR data enhances differentiation between stainless steel and non-metals. The miss classification of cellulose and textiles decreased. But unlike above, adding IR features did not help differentiating between aluminum and stainless steel.

### Discussion

The asymptotic behavior of the solution is examined as part of a convergence analysis and by a repeated cross validation. Based on these experiments, the material specific improvement is assessed using confusion matrices and boxplots.

In summary, IR features as an additional feature enhance differentiation between materials and boost recognition ability. The accuracy of the solution increases significantly when including IR data.

Materials which visually appear to be similar, such as wood and wood imitate, or aluminum and stainless steel were classified and could be differentiated more precisely.

This study uses a SVM to combine learned visual features from a CNN with engineered IR features from thermal imaging. To evaluate the results, the classification is done with and without these features. The aim of this study is not to reach the best possible classification results but to examine whether infrared data helps to increase the recognition accuracy.

Nevertheless the proposed method clearly outperforms previous approaches. It is remarkable that other fusion algorithms leads to poorer classification results than evaluating the data without fusion.

One possibility could be the homogeneity of the respective temperature fields. These appear more material specifically constant and without texture. Feature and image fusion approaches, on the other hand, try to combine characteristic textures.

## Conclusion and outlook

In the age of deep learning, the main challenge in material recognition is not feature engineering but data collection. Nevertheless, it is shown that engineered features based on a physical model can still help to improve the recognition accuracy.

When extending the evaluated electromagnetic spectrum to the IR range, a significant improvement of recognition is possible. With the engineered features, the overall mean accuracy increases by 6 pp. Also, the additional features help to classify materials which visually appear to be the same.

Therefore, over 1100 VIS and IR images were taken from ten material classes in controlled indoor environments. Even if the evaluated temperature distributions, based on the IR emissivity, do not lead to material specific fingerprints, they help differentiating between certain materials when used as an additional feature.

Based on the three research hypotheses, this article provides the following answers:

- (RH 1) Evaluating the IR range additionally to the VIS range has proven to be an effective option to significantly boost the reliability of material recognition for industrial processes.
- (RH 2) Therefore, materials which are broadly applied in industry and have certain similarities in texture and color are evaluated. Additionally, different lighting conditions and recording distances are taken into account.
- (RH 3) The IR features used are material-specific and increase classification accuracy. The proposed sensor fusion algorithm is realized with a SVM which has proven to be a suitable option for this material recognition application.

The results show significant improvements for material recognition. However, it is not examined whether these are the best possible accuracy results. Therefore, an extended training by comparing different pipelines is necessary.

In addition, the database should be extended with more material samples to further investigate the reliability of the results and to increase the generalization ability. While the data of metals seems to appear characteristic, the identification of cellulose and plastic in particular must be backed with additional training samples in order to obtain sound classification results.

## Data Availability

The dataset used and analysed during the current study is available from the corresponding author on reasonable request.
